# From Mosquito Bites to Sexual Transmission: Evaluating Mouse Models of Zika Virus Infection

**DOI:** 10.3390/v13112244

**Published:** 2021-11-08

**Authors:** Elizabeth Balint, Amelia Montemarano, Emily Feng, Ali A. Ashkar

**Affiliations:** McMaster Immunology Research Centre, Department of Medicine, McMaster University, Hamilton, ON L8S 4L8, Canada; balins1@mcmaster.ca (E.B.); montea2@mcmaster.ca (A.M.); fengey@mcmaster.ca (E.F.)

**Keywords:** Zika virus, mouse model, mosquito-borne disease, sexually transmitted infections

## Abstract

Following the recent outbreak of Zika virus (ZIKV) infections in Latin America, ZIKV has emerged as a global health threat due to its ability to induce neurological disease in both adults and the developing fetus. ZIKV is largely mosquito-borne and is now endemic in many parts of Africa, Asia, and South America. However, several reports have demonstrated persistent ZIKV infection of the male reproductive tract and evidence of male-to-female sexual transmission of ZIKV. Sexual transmission may broaden the reach of ZIKV infections beyond its current geographical limits, presenting a significant threat worldwide. Several mouse models of ZIKV infection have been developed to investigate ZIKV pathogenesis and develop effective vaccines and therapeutics. However, the majority of these models focus on mosquito-borne infection, while few have considered the impact of sexual transmission on immunity and pathogenesis. This review will examine the advantages and disadvantages of current models of mosquito-borne and sexually transmitted ZIKV and provide recommendations for the effective use of ZIKV mouse models.

## 1. Introduction

Despite the discovery of Zika virus (ZIKV) nearly 70 years ago, ZIKV emerged in the 2010s as a significant health threat [1]. The most recent outbreak in 2015–2016 in Latin America unveiled several devastating outcomes of ZIKV infection, including Guillain-Barré syndrome (GBS) in adults and ZIKV-induced microcephaly in the developing fetus, resulting in long-term cognitive impairments [1,2,3]. ZIKV is now endemic in many countries in Southeast Asia, Africa, and South America [4]. Several local outbreaks of ZIKV infections have also been reported in India, with concerns that a future ZIKV outbreak could be devastating in densely populated areas of India and other countries [5].

Although ZIKV infections are largely believed to be transmitted via infected *Aedes aegypti* and *A. albopictus* mosquitos, several studies have demonstrated that ZIKV can also be sexually transmitted and is capable of vertical transmission to the developing fetus [1,6,7]. Sexual transmission of ZIKV disproportionately affects women, with approximately 90% of sexually transmitted ZIKV infections being male-to-women transmission [8]. Furthermore, ZIKV RNA has been detected up to 188 days post-infection in semen, presenting a large window of opportunity for sexual transmission [8,9]. Pregnancy is also known to increase susceptibility and adverse outcomes to many viral infections, and these immunological alterations may also increase susceptibility to vaginal ZIKV infections [10,11]. In South America, greater consideration of public health protocols and planned pregnancy during months of lower risk of mosquito-borne infection has been suggested to reduce the risk of harm to the developing fetus [12]. However, the longevity of ZIKV infection in males extends the risk of ZIKV infection beyond the seasonal and geographical range of mosquito-borne transmission. Thus, sexually transmitted ZIKV infections remain a risk in countries where ZIKV is endemic and pose significant risk for microcephaly and other ZIKV-induced harm to the developing fetus.

With ZIKV outbreaks reoccurring every 5–10 years and no vaccines or effective treatments available, ZIKV is likely to result in outbreaks of increasing severity until the development of an effective vaccine. Following the 2016 outbreak, many immunodeficient and immunocompetent mouse models have been used to investigate ZIKV tropism, immunity, and potential treatments or vaccines. However, several questions remain unanswered regarding natural ZIKV infection and the impact of the route of infection on host immune responses and disease outcomes. While several animal models in hamsters, rhesus macaques, and pigtail macaques have been established [13,14,15], mouse models remain the most widely used models of ZIKV infection and have informed the majority of our knowledge of ZIKV-induced disease. In this review, we will examine the current knowledge regarding mosquito-borne vs. sexually transmitted ZIKV infections, examine the advantages and disadvantages of mouse models of ZIKV infection, and discuss how these can be used to answer these timely questions.

## 2. Models of Mosquito-Borne Zika Virus (ZIKV) Infection

Following the 2016 outbreak in Latin America, a mouse model for ZIKV infection was crucial to begin investigating ZIKV-induced immunity and disease. Several models have been established to model mosquito-borne ZIKV infection, including both immunocompromised and immunocompetent mouse models. These mainly employ footpad, subcutaneous, and intraperitoneal injection to mimic vector transmission, with footpad infection being most common [16,17,18,19,20,21,22]. As these routes of infection may elicit different trafficking patterns through the draining lymph nodes, use of footpad infection may elicit more consistent immune responses [23]. It has been well-established that ZIKV and other flaviviruses utilize the viral NS5 protein to bind to and degrade STAT2 proteins downstream of type I interferon receptor (IFNAR) signaling, inhibiting expression of interferon (IFN)-stimulated genes and induction of innate antiviral responses [24,25]. Consequently, ZIKV evades host defense mechanisms and can efficiently replicate in human cells. ZIKV NS5 protein cannot target murine STAT2 to evade IFN signaling, which hinders the use of wild-type (WT) mice in studying ZIKV-induced disease [24,26]. *Ifnar*^−/−^ mouse models and other similarly immunocompromised mouse models, including *Stat2*^−/−^ mice, exhibit enhanced susceptibility to ZIKV infections and remain most frequently used for the study of ZIKV infection and pathogenesis in vivo. Mouse models lacking IFN signalling are more susceptible to ZIKV infection and display higher viral titers and viral persistence in tissues compared to WT mice [16,17,21,27,28,29,30]. As ZIKV infections have been associated with increasing rates of GBS and other neurological disorders, IFNAR-deficient models on a C57BL/6 background have demonstrated ZIKV dissemination to the CNS, accompanied by neurological deficits, and can be useful for studying the mechanism of ZIKV-induced neuropathology [16,18,30,31]. For example, Lazear et al. demonstrated that *Ifnar*^−/−^ and *Irf3*^−/−^*Irf5*^−/−^*Irf7*^−/−^ triple knockout (TKO) mice inoculated subcutaneously with ZIKV sustained higher levels of infection in the testes, spinal cord, and brain compared to WT mice and exhibited signs of neurological disease, such as hind limb weakness and paralysis, starting around 6 days post-infection (dpi). By 10 dpi, mice in both *Ifnar*^−/−^ and TKO groups were either moribund or dead, in contrast to ZIKV-infected WT mice that displayed no signs of disease throughout the duration of the experiment. Similarly, Morrey et al. recently described an aged *Ifnar*^−/−^ mouse model, where infection of 4-month-old mice was not as lethal as infection of younger mice and elicited transient paralysis that may facilitate further investigation of GBS [32].

A129 mice (lacking the type I IFN receptor) and AG129 mice (lacking both type I and type II IFN receptors) on a 129Sv/Ev background have also been used as a model of ZIKV infection with a variety of ZIKV strains [17,27,28,33,34,35]. Despite neuroinvasion by ZIKV in A129 and AG129 mice, neurological outcomes, such as hindlimb paralysis, are less commonly observed in mice of the 129Sv/Ev background compared to reports in C57BL/6 mice. Thus, the mouse strain background is an important factor that must be considered in investigations of immunity during ZIKV infection as well as development of novel vaccines or therapeutics. *Ifnar*^−/−^ mice on both backgrounds have been shown to develop sufficient adaptive immunity, including memory T cells and neutralizing antibodies in response to infection or vaccination, and can be employed to investigate potential vaccines [18,36,37,38,39,40]. However, vaccine-induced reduction in ZIKV viremia seen in A129 and AG129 mice may not necessarily indicate sufficient prevention of CNS invasion and subsequent disease. It is critical that while vaccines and therapeutics may function in A129 or AG129 mice, they should also be evaluated for protection against ZIKV-induced neurological disease in *Ifnar*^−/−^ mice of the C57BL/6 background.

### 2.1. Monoclonal Antibody Blockade of Type I Interferon Receptor (IFNAR) Elicits Similar Clinical Outcomes to IFNAR-Deficient Mice and May Be an Ideal Model for Evaluation of Vaccines

Although *Ifnar*^−/−^ models provide a good basis to study pathogenesis of ZIKV-induced disease, defective innate immune responses may impact the development of adaptive immunity through impaired antigen presentation and B and T cell priming, which may underestimate the efficacy of therapeutic interventions and vaccines that rely on these immune responses. To establish models that better represent human antiviral immune responses, several groups have administered anti-IFNAR monoclonal antibodies (mAbs) to immunocompetent mice as opposed to using transgenic *Ifnar*^−/−^ models [31,39,41,42,43,44]. Smith et al. established a partially immunocompetent model of ZIKV infection that demonstrated severe ZIKV-induced disease, neuroinflammation, and mortality following administration of anti-IFNAR in C57BL/6 WT mice 1 day before ZIKV inoculation [31]. Both subcutaneous and intraperitoneal inoculations led to weight loss, viremia, and neuropathologic changes [31]. These results demonstrated similar neurological disease in mAb treated mice as IFN-deficient models previously mentioned, suggesting antibody blockade against IFNAR may be a suitable model to further study neurological damage caused by ZIKV infections using transgenic mice of the same background. Importantly, antibody blockade has not elicited consistent neurological disease in other studies, perhaps due to incomplete blockade and inability for the antibody to cross the blood–brain barrier, thus the dose and resulting clinical symptoms should be verified before use [16]. Compared to a completely IFN-deficient mouse model, the use of anti-IFNAR antibody blockade allows the mice to maintain normal innate immune responses during the vaccination period until suppression of IFN signalling during the time of ZIKV challenge. However, several studies have demonstrated strong efficacy of candidate vaccine-induced adaptive immunity in the absence of IFNAR signaling for both HSV-2 and ZIKV, suggesting that use of an IFNAR-deficient mouse model may be sufficient to evaluate vaccine strategies against ZIKV [36,37,38,40,45].

### 2.2. Alternative Models to Immunocompromised Mice May Not Accurately Represent Natural Infection

Due to limitations of completely IFNAR-deficient models, some groups have attempted to circumvent IFN-deficiency by use of neonatal models and intracranial injections. Studies have shown that neonatal mice can be infected by ZIKV since their immune system is not yet fully functional and exhibits reduced IFN signaling [44,46,47,48,49]. For example, Manangeeswaran et al. established a model of neonatal infection of WT mice at postnatal day one [46]. While WT mice were susceptible to infection and developed mild neurological disease, the infected neonatal *Ifnar*^−/−^ mice displayed higher viral titers in the CNS, liver, and spleen and developed lethal neurological disease, such as hind limb bilateral paralysis [46]. They also observed differences in immune cell infiltration in the CNS, where WT mice exhibited higher levels of CD8^+^ T cells and *Ifnar*^−/−^ mice displayed increased macrophage and neutrophil infiltration. Interestingly, these results contrast studies in adult IFNAR-deficient mice, where CD8^+^ T cells comprise the majority of infiltrating immune cells [18,19,20,30]. Using immunocompetent neonatal mice may also have its disadvantages, as the immune system is still being developed and may not recapitulate the maturity of immune responses observed in adults [50]. Regardless, neonatal mice may be a viable option for investigating questions regarding neurological disease or testing antiviral drugs without a complete lack of type I IFN signaling capabilities.

**Figure 1:**
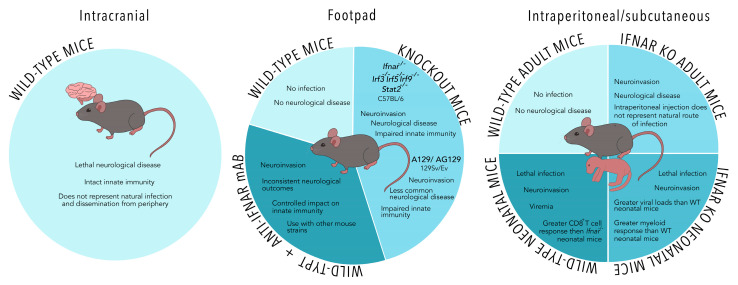
A comparison of models of mosquito-borne Zika virus (ZIKV) infection in non-pregnant mice. IFN-deficient mouse models and the use of anti-IFNAR (type I interferon receptor) monoclonal antibodies are commonly employed to investigate ZIKV infection using subcutaneous, intraperitoneal, or footpad injections as depicted. Footpad infections may be a more representative route to mimic mosquito-borne infection. Intracranial and neonatal models of ZIKV infection bypass the requirement for IFN-deficient transgenic mice but may not represent natural infection of adult humans.

While immunocompetent mouse models have provided critical understanding of ZIKV epitopes and development of effective adaptive immunity during vaccination, they are not capable of modeling ZIKV infection and replication without some form of IFN-deficiency [20,22,29]. Several groups wishing to investigate mechanisms of ZIKV-associated neurological disease and potential therapeutics have opted to employ intracranial inoculation to bypass clearance by the immune system in the periphery before reaching the CNS. Studies using intracranial injections have spanned investigations of T cell and microglia interactions to altered metabolic states in neurons during ZIKV infection [51,52,53,54]. However, evading the peripheral immune system through intracranial infection may drastically alter immune responses in the CNS that can contribute to protection or neuropathology following ZIKV infection. Figueirdo et al. observed ZIKV RNA escape to the periphery following intracranial infection, which has essentially reversed the order of natural ZIKV infection [51]. This reversed order of dissemination does not account for the involvement of peripheral innate and adaptive immune cells in ZIKV dissemination to the CNS or subsequent CNS infiltration and immune-mediated damage following natural infection. Intracranial infection may be beneficial in testing antivirals that can reduce replication in the CNS, but studies of the immune response should be interpreted with caution and confirmed in other models that more closely mimic natural ZIKV infection. Overall, while alternative models to IFNAR-deficient mice can provide helpful insight and understanding of ZIKV infections, the immunocompromised *Ifnar*^−/−^ mouse model remains the model that most closely resembles natural infection via mosquito-borne transmission (Figure 1).

## 3. Models of Sexually Transmitted ZIKV Infection

While vector-borne ZIKV infections are a major concern, ZIKV emerged in the South American epidemic as a novel sexually transmitted virus. With few travel restrictions, many infected individuals returned to their home countries, where they presented symptoms of infection or were suspected to have transmitted the virus via unprotected sexual intercourse [9,55,56]. The spread of ZIKV beyond its vector-restricted geographical range presents a risk for future outbreaks of sexually transmitted ZIKV, and mouse models that consider the impact of sexual transmission are necessary to develop vaccines, therapeutics, and public health strategies to protect women impacted by this disease.

### 3.1. Sexual Transmission May Be More Harmful Than Mosquito-Borne Infection during Pregnancy

The recent ZIKV outbreaks demonstrated the severity of ZIKV infections, as neonates from infected mothers presented with microcephaly and other neurological impairments [2,3,57]. Models of ZIKV infection during pregnancy have been a critical focus since 2016, with significant research directed towards the impact of mosquito-borne ZIKV infection and timing of infection during pregnancy [58,59,60,61,62,63,64,65]. These models have also been used to evaluate vaccines that prevent against ZIKV-induced damage to the developing fetus [37,66,67,68,69]. However, the discovery that ZIKV can be sexually transmitted has complicated efforts to study ZIKV infections of pregnant mice. Although subcutaneous or intraperitoneal injections effectively model mosquito-borne ZIKV, women are also at risk of sexual exposure to ZIKV by infected males [6,7,8,70]. Studies of both subcutaneous/footpad and intravaginal infection have demonstrated that pregnant WT or *Ifnar*^+/*−*^ mice can be infected through both routes, although partial or complete IFN deficiency is necessary for sustained replication [11,58,65].

Studies using vaginally administered ZIKV following mating of uninfected pairs have provided clarity regarding consequences of ZIKV infection at different timepoints during pregnancy [11,71,72]. For example, Yockey et al. show that intravaginal (IVAG) infection at embryonic day 4.5 (E4.5), during early pregnancy, results in reduced fetal weights at E18.5 in both WT and *Irf3*^−/−^*Irf7*^−/−^ breeding pairs [11]. Meanwhile, mating of *Ifnar*^−/−^ dams with a WT sire, intended to resemble the immune status of the human fetus, resulted in resorption of the fetus following IVAG E4.5 infection, and E8.5 IVAG infection significantly reduced fetal weight at E18.5 [11]. However, this model does not reflect simultaneous conception and ZIKV infection by infected males. In *Ifnar*^−/−^ mice, Uraki et al. demonstrated male-to-female transmission resulting in simultaneous infection and pregnancy and observed fetal growth restriction and ocular deformities at E18.5 [73]. Similarly, Winkler et al. established a mouse model of sexual transmission from anti-IFNAR treated *Rag1*^−/−^ (AIR) mice to *Ifnar*^−/−^ or AIR dams [43]. Mating of AIR sires and dams resulted in vertical transmission measured by infection of the placenta and fetus, although clinical outcomes of fetal development were not assessed. This study further demonstrated the ability to use antibody blockade with a variety of transgenic mice, which may be an ideal method since it uses partial IFNAR blockade for closer resemblance to human infection and does not require extensive breeding.

It has been suggested that pregnancy may impair development of CD4^+^ and CD8^+^ T cell responses following ZIKV infection, although neutralizing antibody production is not significantly altered [39]. However, Duggal et al. did not observe any significant difference in survival between infected pregnant and non-pregnant AG129 mice, suggesting that the timing of infection during pregnancy may determine the clinical relevance of this impairment [35]. However, this research group also observed greater dissemination of infectious ZIKV to the uterus in pregnant females compared to non-pregnant females, indicating some differences in susceptibility to infection in pregnant individuals that may be mediated by decreased cellular immunity [35,74]. Additionally, Duggal et al. observed increased dissemination to the fetus following sexual transmission compared to mosquito-borne infection, but they did not assess clinical outcomes of disease in the fetus [74]. Further research is necessary to conclude if the route of infection does differ in its impact on fetal development. Consequently, models of varying routes and timepoints of infection of pregnant mice must be considered when testing vaccines and therapeutics to ensure optimal efficacy in preventing all causes of infection.

### 3.2. Models of Sexual Transmission in Non-Pregnant Mice Demonstrate Different Kinetics of ZIKV Infection and Disease

While the vast majority of research investigating ZIKV as an STI in mouse models has been focused on pregnant females, few have investigated how sexual transmission of ZIKV impacts ZIKV dissemination and disease outcomes in non-pregnant mice. Soon after the ZIKV outbreak in 2016, Tang et al. reported the use of diestrus-induced type I and type II IFN receptor-deficient AG129 mice to model IVAG ZIKV infection [75]. They further demonstrated the necessity of IFNAR deficiency on myeloid-derived cells on a C57BL/6 background for IVAG-infected mice to establish a systemic ZIKV infection [75]. Yockey et al. similarly demonstrated that IVAG-infected *Ifnar*^−/−^ mice on a C57BL/6 background succumb to ZIKV-induced hindlimb paralysis and weight loss [11]. Administration of a monoclonal blocking antibody against IFNAR renders mice infected via intravaginal inoculation or sexual transmission susceptible to infection [36,43]. As previously described, anti-IFNAR mAb treatment may not represent a complete blockade and does not always result in neurological disease as is observed in *Ifnar*^−/−^ mice. These anti-IFNAR treatments may facilitate investigation of sexually transmitted ZIKV kinetics and dissemination in mouse models lacking lymphocytes or other components of the immune system, as described by Winker et al. with footpad infections [39].

Immunocompetent mice have also been employed to investigate how immune responses in the vaginal tract may differ from other routes of infection. Several studies have indicated that WT mice are susceptible to IVAG ZIKV infection during progesterone-induced diestrus, but these mice are incapable of developing a systemic ZIKV infection due to their functioning type I IFN signaling [11,76]. Khan et al. demonstrated a dampened immune response in the vaginal tract of ZIKV-infected WT mice, likely due to low expression of the pattern recognition receptors RIG-I, MDA5, TLR3, and TLR7 in the lower female reproductive tract compared to the upper female reproductive tract and the iliac lymph node [76,77]. This results in minimal type I IFN production and sustained but low detection of ZIKV RNA for 3 days [76]. However, the use of qPCR to detect ZIKV RNA cannot convincingly determine if this sustained, but low, viral replication produces infectious ZIKV particles. Because the authors used an immunocompetent mouse model, they were unable to assess how sustained replication in the vaginal tract impacts dissemination of infectious ZIKV particles or disease outcomes. Furthermore, although they demonstrate a delay in CD8^+^ T cell priming and control of LCMV replication in the vaginal tract, the impact of dampened adaptive immunity on ZIKV replication and neurological disease cannot be assessed using WT mice [76].

Thus far, one research group has attempted to investigate how the route of ZIKV infection might impact ZIKV viremia or dissemination to other tissues. Duggal et al. show that sexual transmission of ZIKV in AG129 mice resulted in more rapid and severe weight loss and shorter mean survival compared to mice subcutaneously or intravaginally infected [74]. Interestingly, non-pregnant IVAG and sexually infected mice appeared to exhibit worse survival than pregnant mice, although this was not directly compared. They further observed that IVAG infection resulted in a 4-day delay in viremia compared to subcutaneous infection or sexual transmission in both pregnant and non-pregnant mice, although this may be due to inconsistent or impaired IVAG infection due to the use of mice at random stages of the estrous cycle [74]. They further demonstrate that sexual transmission in non-pregnant female mice resulted in infection of the uterus and ovary at the time of euthanasia, while they observed little to no infection in these organs in non-pregnant mice with the other routes of infection. These results should be interpreted with caution, as the mice that were subcutaneously and intravaginally infected also survived much longer than the sexually infected mice and may have cleared the infection by that time. Although the impact of the route of infection on neurological disease cannot be assessed in this model, these results suggest that the route of infection may impact ZIKV kinetics and disease in non-pregnant mice and may help us to better understand how to combat sexually transmitted ZIKV and associated disease in women.

**Figure 2:**
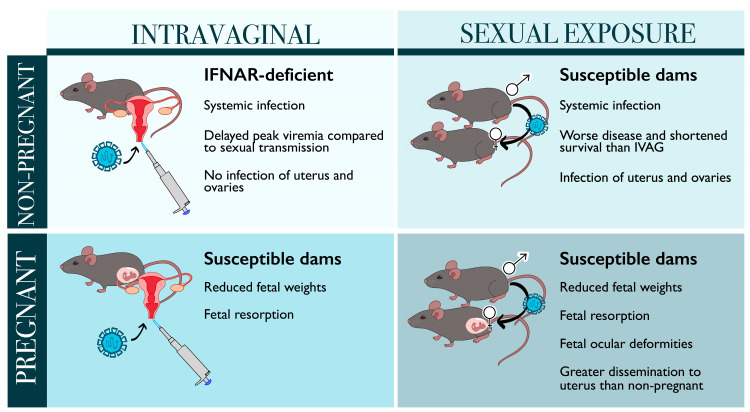
Models of sexually transmitted ZIKV infections in both pregnant and non-pregnant mice. Sexual transmission results in worse survival and greater dissemination to the uterus and ovaries in non-pregnant females compared to intravaginally infected (IVAG) mice. Both intravaginal infection and sexual exposure can impair fetal development, but these clinical outcomes have not been directly compared.

As described, Duggal et al. demonstrated that there may be some differences in kinetics and dissemination of ZIKV between the sexual exposure and IVAG infection models used to represent sexually transmitted ZIKV infections in both pregnant mice and their fetuses as well as non-pregnant mice [74]. Several studies have established models of sexually transmitted ZIKV by intraperitoneal or subcutaneous/footpad-infected males deficient in IFN signaling and mating them with uninfected dams of various genetic backgrounds [35,43,73,74]. While many of these studies were previously described in the context of pregnancy, many have also used these models to investigate the effects of semen and infected sperm that may be more representative of natural infection. Clancy et al. have established in A129 and AG129 mice that ZIKV infects all parts of the male reproductive system, with titers of 10^3^ PFU per ejaculate observed, and vaginal inoculation of female mice with epididymal flush or seminal plasma results in ZIKV infection and viremia [35,78,79,80]. A129 mice may be a more suitable model than AG129 mice to recapitulate ZIKV infection of the human male reproductive tract, as these mice exhibited mild lesions similar to humans and histologically normal sperm for studying sexual transmission [79]. Furthermore, the differences in kinetics and dissemination of ZIKV following sexual exposure vs. IVAG infection may be attributed to micro-abrasions facilitating more rapid viral dissemination or components of ejaculate, including semen, that may alter inflammatory responses and promote ZIKV replication, as seen with other viruses [81,82]. However, sexual exposure models remain difficult to control, as mating infected males with naïve dams or inoculation with seminal plasma results in inconsistent viral titers for inoculation that may influence ZIKV kinetics and immune responses. While IVAG inoculation controls for viral titer and is unlikely to alter inflammation in the vaginal tract, this model may not capture all aspects of sexual exposure that may influence ZIKV replication and dissemination. Overall, each model possesses several advantages and disadvantages that should be considered in future investigation of sexually transmitted ZIKV infections (Figure 2).

## 4. Looking into the Crystal Ball: The Future of ZIKV Mouse Models

Although it has been suggested that kinetics and dissemination of ZIKV differ in IVAG and sexually transmitted models of ZIKV compared to subcutaneous/footpad or intraperitoneal injections, it remains unclear why this occurs and how it impacts development of ZIKV-induced disease in non-pregnant mice. Despite their limitations, immunodeficient mice, deficient in type I IFN receptor signaling or select ISGs, will be necessary to investigate the clinical relevance of differing routes of ZIKV infection. Antibody blockade of IFNAR offers flexibility to investigate ZIKV infection kinetics and disease in other models rather than requiring additional breeding to establish double knockout mice.

The mechanism of dissemination of ZIKV from the primary site of infection, including the role of semen and the impact of sexual transmission in ZIKV dissemination, remain poorly understood. Although humanized mouse models have only recently been established for ZIKV infection, they further corroborate evidence that immune cells are required for dissemination and development of systemic ZIKV infection following subcutaneous or intraperitoneal infection [83,84]. Humanized mouse models may provide a new avenue to investigate the role of the immune system in ZIKV dissemination from the female reproductive system, assuming that this is largely mediated by infected immune cells. However, these models cannot facilitate infection of structural cells with functional IFN signaling. Studies in humanized mice should be carefully compared to current immunodeficient models and knowledge from studies using human samples to evaluate their accuracy in depicting human ZIKV infection, particularly for sexually transmitted infection models. A more physiologically relevant model may be transgenic human STAT2-expressing (hSTAT2) mice. Gorman et al. established a model of hSTAT2 mouse infection, rendering the mouse susceptible to ZIKV evasion of type I IFN signaling, and observed increased ZIKV replication and mortality in 3-week-old mice infected with a mouse-adapted African ZIKV Dakar strain first isolated in 1984 [85]. Several studies have indicated differences in clinical disease outcomes of African strains and those isolated in the French Polynesian and South American epidemics [30,48,49,85]. Use of this hSTAT2 mouse model with more relevant strains from the recent South American outbreak or those currently circulating in ZIKV-endemic countries may provide the most accurate representation of human ZIKV-induced disease and open avenues to investigate both mosquito-borne and sexually transmitted ZIKV infections as well as vaccine and antiviral development.

## 5. Conclusions

Transgenic IFNAR-deficient mouse models remain the most employed model of ZIKV infection, despite their limitations. Several alternatives, including use of anti-IFNAR antibodies, immunocompetent mice, neonatal mice, and intracranial injections have been investigated but possess their own limitations that must be carefully considered. Advances in humanized mouse models of ZIKV may provide the ability to answer specific questions regarding ZIKV infection of hematopoietic cells and their role in dissemination of ZIKV. Use of human STAT2 transgenic mice with clinically relevant ZIKV isolates should be further investigated, as they may represent the most relevant model of human infection thus far.

Although significant research focus has been channeled towards understanding mosquito-borne ZIKV and establishing mouse models of ZIKV infection, our understanding of sexually transmitted ZIKV infections remains limited. In the context of pregnancy, we are only beginning to understand the effects of simultaneous conception and sexual transmission of ZIKV in fetal development. With the potential for future outbreaks of ZIKV, an STI that breaks the geographical confines of vector-borne infection, it is critical to consider sexual transmission in mouse models of ZIKV infection and develop vaccines and treatments that will prevent against both mosquito-borne and sexually transmitted ZIKV.
